# The complete mitochondrial genome of *Pheidole nodus* (Smith, 1874) (Hymenoptera: Formicidae)

**DOI:** 10.1080/23802359.2022.2047118

**Published:** 2022-03-06

**Authors:** Yu Sang, Ru-Yi Yin, Yi Luo, Zhao-Min Zhou

**Affiliations:** a Key Laboratory of Southwest China Wildlife Resources Conservation (Ministry of Education), China West Normal University, Nanchong, China; b Key Laboratory of Environmental Science and Biodiversity Conservation (Sichuan Province), China West Normal University, Nanchong, China

**Keywords:** *Pheidole nodus*, Myrmicinae, mitochondrial genome, phylogenetic analysis

## Abstract

*Pheidole nodus* (Smith, 1874) belongs to a famously hyperdiverse and ecologically dominant ant genus. The mitochondrial genome of *P. nodus* is 15,579 bp in length, and the overall base composition is 78.6% AT. It includes 13 protein-coding genes, 2 ribosomal RNA genes, 22 transfer RNAs, and a control region. Phylogenetic trees show that *P. nodus* is more closely related to *Wasmannia* than to *Atta*. These sequence data will play an important role in the investigation of the phylogenetic relationships and taxonomy of the group Attini.

*Pheidole*, diet generalists of approximately 1200 known ant species (Bolton 2020), are found in most temperate and tropical biomes and on every continent except Antarctica and are particularly dominant in tropical habitats (Economo et al. 2015). Despite being the largest of all genera of plants and animals (Wilson 2003), *Pheidole* currently lacks a completely sequenced mitochondrial genome. Based on nuclear gene fragments, *Pheidole* is within the Attini group with *Allomerus*, *Anisopheidole*, *Blepharidatta*, *Cephalotes*, *Diaphoromyrma*, *Lachnomyrmex*, *Lenomyrmex*, *Machomyrma*, *Ochetomyrmex*, *Procryptocerus*, *Tranopelta* and *Wasmannia*, as well as all the fungus-growing ants (e.g. *Atta*) (Ward et al. 2015). However, based on the complete mitochondrial genome, the Attini group is polyphyletic because *Wasmannia* was shown to be closely related to the Solenopsidini group with strong support (Park et al. 2020; Yin et al. 2022). *Pheidole nodus* is widely distributed in eastern Asia, often occurring from open lands to relatively developed forests, and nests in the soil. Here, to better understand the phylogenetic relationship, we present the complete mitochondrial genome of *Pheidole nodus* as the first mitogenome of the genus *Pheidole*.

The specimens of *Pheidole nodus* workers were collected from a well-established colony in Nanchong City (30°49′25.30ʺN, 106°3′49.87ʺE), China, in October 2020. All protocols in the sample collection were reviewed and approved by the Research Ethics Committee of China West Normal University (Approval reference: CWNU2020D002). These specimens were preserved at −80 °C at the Key Laboratory of Southwest China Wildlife Resources Conservation, China West Normal University (www.cwnu.edu.cn; contact person: Yi LUO, v_luoyi@126.com), after morphological identification under voucher number NCPN202010. Total genomic DNA was extracted and sequenced with the Illumina Novaseq sequencing platform with 150 bp paired-end reads by Shanghai Personal Biotechnology Co. Ltd, China. The genome de novo assembly was carried out with the software A5-miseq pipeline (Coil et al. 2015) and SPAdes V.3.14.1 (Bankevich et al. 2012). The MITOS Web Server (Bernt et al. 2013) was used for mitogenome annotation. The complete mitochondrial genome sequence information was deposited in GenBank under accession number MW429351.

The mitochondrial genome of *P. nodus* is 15,579 bp long and consists of 13 protein-coding genes (PCGs), 2 rRNAs, 22 tRNAs, and a control region. Its GC ratio is 21.4%. All PCGs used ATN (five ATG, four ATT and four ATA) as the start codon and TAA or TAG as the stop codon. The tRNA sizes range from 53 to 71 bp and are similar to those of other ants (approximately 54–90 bp) (Idogawa, 2021). Four PCGs (ND5, ND4, ND4L, and ND1) and ten tRNAS (tRNA-Val, Gln, Cys, Cys, Phe, His, Leu, Pro, and rRNA-Leu, Ser) are encoded by the majority strand (J-strand), while the others are located on the minority strand (N-strand). The gene order of *P. nodus* has two rearrangements compared to those of most Myrmicinae species: *inversions between trnS1 and ND5* (Myrmicinae*, trnE- trnF; P. nodus, trnF- trnE*) and *ND* and *ND6* (Myrmicinae, *trnT*- *trnP*; *P. nodus, trnP-trnT*). (Babbucci et al. 2014; Vieira and Prosdocimi 2019).

Thirteen PCGs and two rRNA genes from 29 ants, including *P. nodus* and an outgroup species, *Apis mellifera ligustica*, were aligned using MAFFT 7.450 (Katoh and Standley 2013) and concatenated for phylogenetic purposes in PhyloSuite v1.2.2 (Zhang et al. 2020). The GTR + F + I model was determined as a best-fit model by ModelFinder (Kalyaanamoorthy et al. 2017). Bootstrapped maximum likelihood, neighbor joining, and Bayesian inference trees were constructed using MEGA X (Kumar et al. 2018) and MrBayes 3.2.6 (Ronquist et al. 2012). The phylogenetic trees showed that Crematogastrini and Attini are polyphyletic with low support values (Figure 1), which is congruent with the result based on the complete mitochondrial genome (Park et al. 2020; Yin et al. 2022) but disagrees with that based on nuclear gene fragments (Ward et al. 2015). Furthermore, *Wasmannia* showed a closer relationship to *Pheidole* than to *Atta*, which will aid our understanding of the phylogenetic relationship and taxonomy of the group Attini..

**Figure 1.**
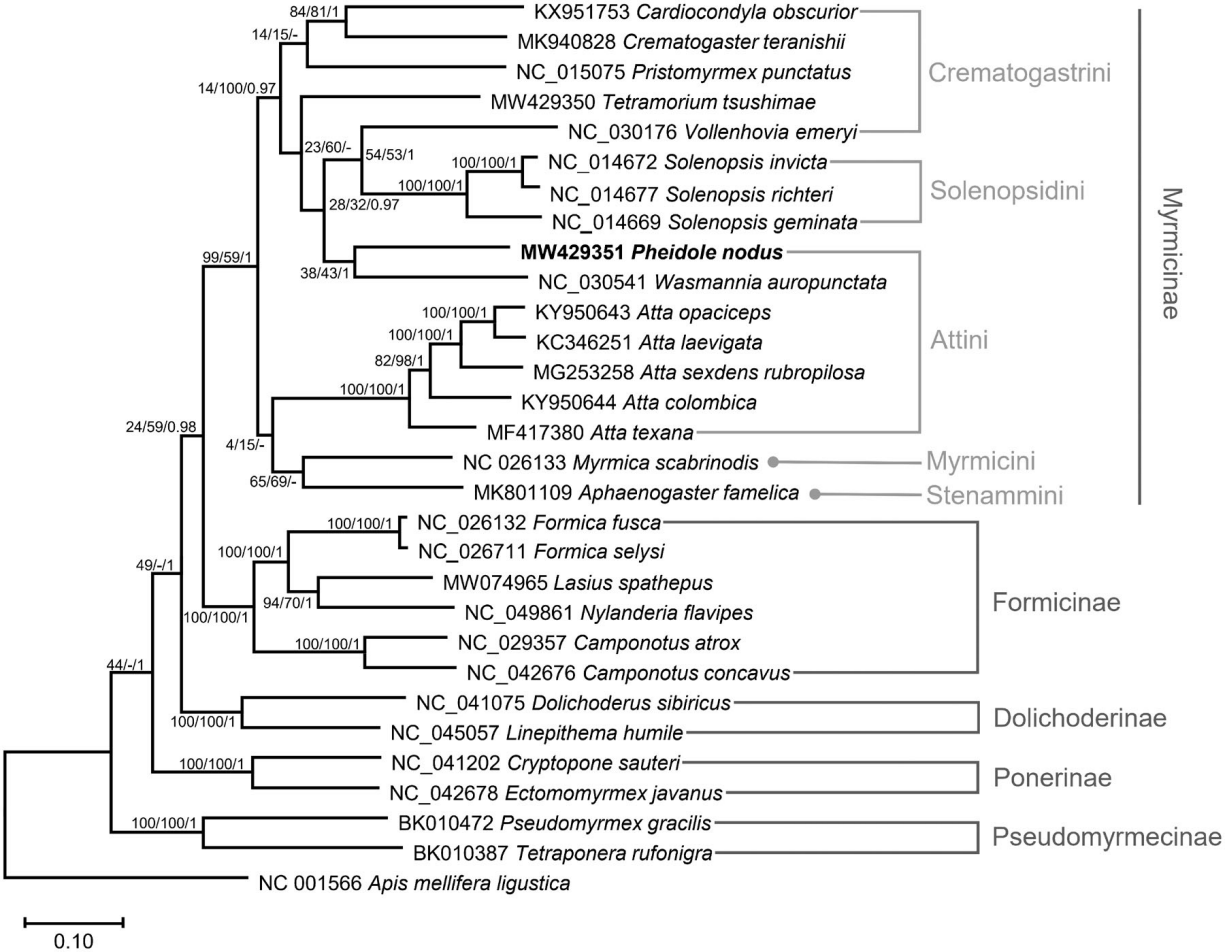
Maximum likelihood phylogenetic tree based on the concatenated PCGs and 2 rTNA genes of 30 Hymenoptera species (29 ants and one bee). Maximum likelihood and Bayesian inference phylogenetic trees were topologically identical. The numbers at the nodes indicate bootstrap support values of maximum likelihood and neighbor-joining trees and posterior probabilities of the Bayesian inference tree.

## Data Availability

The data that support the findings of this study are openly available in GenBank of NCBI at https://www.ncbi.nlm.nih.gov, reference number MW429351. The associated BioProject, SRA, and Bio-Sample numbers are PRJNA772614, SRR16529670 and SAMN22402153, respectively.
